# Protocol for assessing pharmacokinetics and pharmacodynamics of human CAR-NKT cells in humanized mouse models using bioluminescence imaging

**DOI:** 10.1016/j.xpro.2025.103957

**Published:** 2025-07-19

**Authors:** Zibai Lyu, Yan-Ruide Li, Lili Yang

**Affiliations:** 1 Department of Microbiology, Immunology & Molecular Genetics, University of California, Los Angeles, Los Angeles, CA 90095, USA; 2 Department of Bioengineering, University of California, Los Angeles, Los Angeles, CA 90095, USA; 3 Molecular Biology Institute, University of California, Los Angeles, Los Angeles, CA 90095, USA; 4 Eli and Edythe Broad Center of Regenerative Medicine and Stem Cell Research, University of California, Los Angeles, Los Angeles, CA 90095, USA; 5 Jonsson Comprehensive Cancer Center, David Geffen School of Medicine, University of California, Los Angeles, Los Angeles, CA 90095, USA; 6 Parker Institute for Cancer Immunotherapy, University of California, Los Angeles, Los Angeles, CA 90095, USA; 7 Goodman-Luskin Microbiome Center, University of California, Los Angeles, Los Angeles, CA 90095, USA

**Keywords:** Cancer, Immunology, Microscopy, Model Organisms, Biotechnology and bioengineering

## Abstract

Human invariant natural killer T (NKT) cells exhibit strong tumor-killing ability and bridge innate and adaptive immunity. Peripheral blood mononuclear cell (PBMC)-derived chimeric antigen receptor (CAR)-engineered NKT (CAR-NKT) cells show potent anti-tumor activity. Here, we present a protocol for assessing the pharmacokinetics and pharmacodynamics (PK/PD) of PBMC-derived CAR-NKT cells in humanized mouse models using *in vivo* bioluminescence imaging (BLI). We describe steps for evaluating CAR-NKT cell distribution, persistence, and tumor infiltration across tumor-free and tumor-bearing models to support CAR-NKT cell therapy development.

For complete details on the use and execution of this protocol, please refer to Li et al.^1^^,^^2^

## Highlights

• Procedure to generate CAR-NKT cells from healthy donors with high yield and purity
• Instructions to study human CAR-NKT cell behavior using xenograft mouse models
• Steps to assess the *in vivo* pharmacokinetics and pharmacodynamics of CAR-NKT cells

## Abstract (editor highlights)

Publisher’s note: Undertaking any experimental protocol requires adherence to local institutional guidelines for laboratory safety and ethics.

## Graphical abstract

**Figure:**
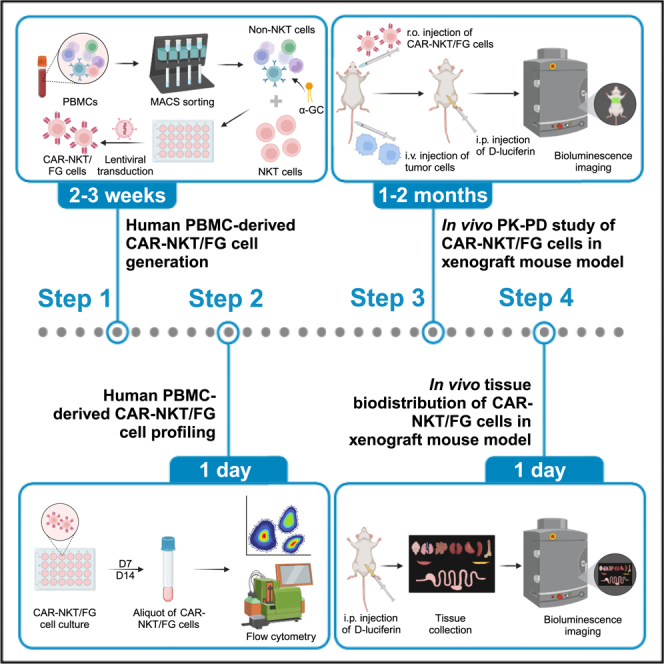

## Before you begin

This protocol outlines the procedures for assessing PK/PD of PBMC-derived CAR-NKT cells in humanized xenograft mouse models using BLI. NKT cells possess strong cytotoxic potential, effective tumor infiltration ability, and capabilities in connecting innate and adaptive immunity.^3^ CAR-NKT cells have shown significant anti-tumor efficacy with minimal toxicity and reduced cytokine release syndrome (CRS) in preclinical and clinical studies.^1^^,^^4^^,^^5^ The protocol specifically focuses on producing B-cell maturation antigen-specific CAR-NKT cells from PBMCs (referred to as BCAR-NKT cells) and assessing their biodistribution, persistence, and tumor-targeting behavior in NSG mice bearing human multiple myeloma (MM) xenografts. Bioluminescence imaging is also applied to analyze their tissue-level localization. The study concentrates on both MM.1S tumor-bearing and tumor-free mice to further the improvement of CAR-NKT-based treatments.

***Note:*** Beyond evaluating PK/PD of CAR-NKT cells, this protocol enables assessment of additional cell-based therapies, including CAR-engineered natural killer (CAR-NK) cells and CAR-engineered gamma delta T (CAR-γδ T) cells. While this study emphasizes testing in tumor-free and human MM xenograft NSG models, researchers can also apply it to examine other hematologic cancers such as AML and solid tumors like melanoma and lung cancer.

### Institutional permissions

Peripheral blood mononuclear cells (PBMCs) from healthy human donors were obtained from the UCLA Center for AIDS Research (CFAR) Virology Core Laboratory under protocols approved by the UCLA Institutional Review Board (IRB). All donors provided informed consent, and samples were de-identified in accordance with federal and state privacy regulations.

All mouse studies were conducted under protocols approved by the UCLA Animal Research Committee (ARC) and performed in specific pathogen-free conditions at UCLA’s Division of Laboratory Animal Medicine (DLAM). Investigators aiming to reproduce this work must obtain appropriate ethical approvals from their own institutions.

## Key resources table

**Table:** 

REAGENT or RESOURCE	SOURCE	IDENTIFIER
**Antibodies**		

Anti-human CD3 (clone HIT3a, 1:500 dilution)	BioLegend	CAT#300329, RRID: AB_10552893
Anti-human CD4 (clone OKT4, 1:400 dilution)	BioLegend	CAT#317414, RRID: AB_571959
Anti-human CD8 (clone SK1, 1:300 dilution)	BioLegend	CAT#344714, RRID: AB_2044006
Anti-human CD69 (clone FN50, 1:50 dilution)	BioLegend	CAT#310906, RRID: AB_314840
Anti-human BCMA (clone 19F2, 1:50 dilution)	BioLegend	CAT#357506, RRID: AB_2562888
Anti-human CD1d (clone 51.1, 1:50 dilution)	BioLegend	CAT#350308, RRID: AB_10642829
Streptavidin (1:1,000 dilution)	BioLegend	CAT#405207
Anti-human invariant NK T cell (clone 6B11, 1:10 dilution)	BD Biosciences	CAT#552825, RRID: AB_394478
Goat anti-mouse IgG F(ab’)2 secondary antibody, biotin (1:50 dilution)	Thermo Fisher Scientific	CAT#31803, RRID: AB_228311

**Bacterial and virus strains**		

Lenti/BCAR-FG	This paper	N/A

**Biological samples**		

Human peripheral blood mononuclear cells (PBMCs)	UCLA	N/A

**Chemicals, peptides, and recombinant proteins**		

a-Galactosylceramide (KRN7000)	Avanti Polar Lipids	SKU#867000P-1mg
Recombinant human IL-7	PeproTech	CAT#200–07
Recombinant human IL-15	PeproTech	CAT#200–15
RPMI1640 cell culture medium	Corning Cellgro	CAT#10-040-CV
Fetal bovine serum (FBS)	Sigma	CAT#F2442
MACS BSA stock solution	Miltenyi	CAT#130-091-376
autoMACS rinsing solution	Miltenyi	CAT#130-091-222
Penicillin-Streptomycin-Glutamine (P/S/G)	Gibco	CAT#10378016
Penicillin: streptomycin (pen:strep) solution (P/S)	Gemini Bio-products	CAT#400–109
MEM non-essential amino acids (NEAA)	Thermo Fisher Scientific	CAT#11140050
HEPES buffer solution	Gibco	CAT#15630080
Sodium pyruvate	Gibco	CAT#11360070
Phosphate-buffered saline (PBS) pH 7.4 (1×)	Gibco	CAT#10010–023
Beta-Mercaptoethanol	Sigma	SKU#M6250
Normocin	InvivoGen	CAT#ant-nr-2
Trypan blue solution, 0.4%	Thermo Fisher Scientific	CAT#15250061
Fixable Viability Dye eFluor506 affymetrix	eBioscience	CAT#65-0866-14
D-luciferin	Caliper Life Science	CAT#XR-1001

**Critical commercial assays**		

TransIT-Lenti Transfection Reagent	Mirus Bio	CAT#MIR 6600
Amicon Ultra-15 centrifugal filter unit	MilliporeSigma	CAT#UFC910024
Cryostor cell cryopreservation media	Sigma	CAT#C2874-100ML
Anti-iNKT MicroBeads, human	Miltenyi	CAT#130-094-842

**Experimental models: Cell lines**		

Human multiple myeloma cell line MM.1S	ATCC	CAT#CRL-2974

**Experimental models: Mice strains**		

NOD.Cg-Prkdc^scid^ Il2rg^tm1Wjl^/SzJ (NSG) mice (6- to 10-week-old female)	UCLA	N/A

**Recombinant DNA**		

Vector: parental lentivector pMNDW	Giannoni et al.^6^; Lan et al.^7^	N/A

**Software and algorithms**		

FlowJo Software 9	FlowJo	https://www.flowjo.com/solutions/flowjo/downloads
Prism 8	GraphPad	https://www.graphpad.com/scientific-software/prism/
Aura Imaging Software	Spectrum Instruments Imaging	https://spectralinvivo.com/software/

**Other**		

Exel International Insulin syringes with fixed needles	Exel International	CAT#76290-402
MACSQuant analyzer 10 flow cytometer	Miltenyi Biotec	CAT#130-096-343
Drummond Portable Pipet-Aid XP pipet controller	Fisher Scientific	CAT#13-681-06
Corning CoolCell FTS30 freezing container	Corning	CAT#CLS432008
Corning 2 mL internal threaded polypropylene cryogenic vial	Corning	CAT#430488

## Materials and equipment

### C10 medium

To prepare C10 medium for culturing T or NKT cells, combine the following components: 100 mL fetal bovine serum (FBS), 10 mL Penicillin-Streptomycin-Glutamine (P/S/G, 100×), 10 mL MEM non-essential amino acids (100×), 10 mL HEPES buffer (1 M), 10 mL sodium pyruvate (100 mM), 10 mL 5 mM β-mercaptoethanol, and 2 mL Normocin (500×). Add these to 848 mL of RPMI to reach a final volume of 1000 mL. Mix the reagents in a sterile 1-L bottle. Use a 0.22 μm filter to sterilize the medium into the bottle. Store the final C10 medium at 4°C in a designated tissue culture refrigerator. The medium remains stable for up to one month.

**Table:** 

Component	Final concentration	Volume (mL)
RPMI	N/A	848
FBS	10%	100
P/S/G (100×)	1×	10
MEM NEAA (100×)	1×	10
HEPES Buffer Solution (1 M)	10 mM	10
Sodium Pyruvate (100 mM)	1 mM	10
β-ME (5 mM)	50 μM	10
Normocin (500×)	1×	2

***Note:*** This C10 media can be used to culture human T cells, NKT cells, MAIT cells, and γδ T cells.

### R10 medium

To prepare R10 medium for culturing suspension tumor cells, thaw 100 mL FBS, 10 mL P/S/G (100×), and 2 mL Normocin (500×) in a 37°C water bath. Measure 888 mL of RPMI medium separately and combine with the thawed components to make a final volume of 1000 mL R10 medium. Refer to the table below for detailed volumes. Use a sterile 1-L autoclaved bottle as the container, and attach a 0.22 μm filter top to filter the medium. Store the R10 medium at 4°C in the tissue culture fridge. It remains stable for up to one month.

**Table:** 

Component	Final concentration	Volume (mL)
RPMI	N/A	888
FBS	10%	100
P/S/G (100×)	1×	10
Normocin (500×)	1×	2

***Note:*** This R10 media can be used to culture tumor cell lines such as human MM cell line MM.1S and human lung cancer cell line H226.

## Step-by-step method details

### Generation of human PBMC-derived BCAR-NKT/FG cells

**Timing: 2–3 weeks**

This protocol describes the generation of firefly luciferase and enhanced green fluorescence protein (FG)-labeled BCAR-NKT (BCAR-NKT/FG) cells derived from PBMCs (Figure 2A). These cells are used to assess PK/PD in humanized NSG mouse models. The method can be adapted to generate other antigen-specific CAR-NKT cells.

1. Preparation of α-GalCer (αGC) (Avanti Polar Lipids, cat. no. 867000) solution.
  a. Preheat a water bath to 80°C and a sonicator to 50°C.
  b. Allow an αGC powder vial to equilibrate to 20°C–25°C.
  c. Dissolve αGC in 1 mL DMSO, invert to mix.
    ***Note:*** Solution should appear cloudy.
  d. Wrap vial cap in parafilm.
  e. Set vial in foam float and heat at 80°C for 10 min.
  f. Sonicate for 10 min.
  g. Vortex for a full 2 min.
    ***Note:*** Solution should now be clear.
  h. Aliquot αGC into glass vials at 25 μL or 60 μL per vial.
  i. Dilute aliquot with at last 200 μL of pre-warmed C10 medium.
    **CRITICAL:** C10 medium should be pre-warmed in 37°C water bath prior to use.
    ***Note:*** Remaining aliquots should be stored in −20°C freezer. Aliquots removed from the freezer should be heated in 80°C water bath for 5 min, sonicated in sonicator for 5 min, and vortexed for a full 60 s before being diluted in pre-warmed C10 medium.
  j. Sonicate for 5 min.
  k. Vortex for a full 60 s.
  l. Add to pre-warmed C10 medium (60 μL aliquot into 12 mL or 25 μL aliquot into 5 mL).
    ***Note:*** This yields 5 μg/mL αGC media.
2. Isolation and enrichment of NKT cells from PBMCs using Anti-iNKT MicroBeads (Miltenyi Biotec, cat. no. 130-094-842), and MACS sorting.
  a. Rapidly thaw freshly isolated human PBMCs in a 37°C water bath.
  b. Transfer cells into a 50-mL conical tube containing 10 mL of pre-warmed C10 medium.
  c. Count live cells using trypan blue exclusion with a hemacytometer.
  d. Centrifuge at 300 × *g* for 5 min and carefully discard the supernatant.
  e. Resuspend the pellet in 400 μL of MACS buffer per 10^8^ cells.
  f. Add 100 μL of Anti-iNKT MicroBeads per 10^8^ total cells.
  g. Mix gently and incubate for 15 min in the dark at 2°C–8°C.
  h. Wash by adding 1–2 mL of MACS buffer per 10^8^ cells, centrifuge at 300 × *g* for 10 min, and aspirate the supernatant.
  i. Resuspend up to 10^8^ cells in 500 μL of MACS buffer.
  j. Insert an LS column into a magnetic MACS Separator.
  k. Pre-rinse the column with 3 mL of MACS buffer.
  l. Load the labeled cell suspension onto the column and collect the flow-through containing unlabeled cells.
  m. Wash the column three times with 3 mL of MACS buffer.
  n. Place the column over a 15-mL conical tube.
  o. Add 5 mL of MACS buffer to the column and immediately elute the retained NKT cells by firmly pushing the plunger into the column.

***Note:*** 5 × 10^8^ PBMCs normally yield 0.5–2 × 10^6^ NKT cells. Donor variability and freeze-thaw cycles can impact yield and purity, which can result in differences in cell recovery and purity. To improve NKT cell purity, perform a second magnetic separation using a fresh column to enrich the eluted fraction. Troubleshooting 1*.*

3. Stimulation of enriched NKT cells with donor-matched irradiated αGC-loaded PBMCs.
  a. Centrifuge the negative fraction at 300 × *g* for 5 min and aspirate the supernatant.
  b. Resuspend cell pellet in 5 mL C10 medium with 5 μg/mL αGC (1–2 × 10^8^ cells/5 mL medium) and incubate at 37°C for 1 h.
    **CRITICAL:** Freeze down the remaining negative portion for future use.
  c. Irradiate cells at 6000 rads.
  d. Determine the cell numbers of the irradiated αGC-loaded PBMCs and the labeled NKT cells using a hemacytometer.
  e. Transfer all NKT cells and the same number of αGC-loaded PBMCs (1:1 ratio) to a 15-mL conical tube.
  f. Centrifuge cells at 300 × *g* for 5 min and aspirate the supernatant.
  g. Resuspend the pellet in C10 medium and plate the cells at 2 × 10^6^ cells in 2 mL per well in a 24-well plate.
  h. Supplement the culture with IL-7 and IL-15 to achieve a final concentration of 10 ng/mL.
  i. Continue the cell culture for the next 7 days.
    **CRITICAL:** Add media containing IL-7 and IL-15 (10 ng/mL) and split the culture upon reaching saturation.
    ***Note:*** On Day 2, proceed to lentiviral transduction.
    ***Note:*** Cells expand 5–10 fold and reach ∼80% NKT in the first week. Troubleshooting 2*.*
4. Lentiviral transduction Troubleshooting 3.
  a. On Day 2, transfer all cells to a 15-mL conical tube and live cells using trypan blue exclusion with a hemacytometer.
  b. Centrifuge at 300 × *g* for 5 min and completely remove the supernatant.
  c. Resuspend the cell pellet in C10 medium and plate at 2 × 10^6^ cells in 2 mL per well in a 24-well plate.
  d. Supplement cultures with IL-7 and IL-15 to a final concentration of 10 ng/mL each.
  e. Add 50–70 μL of Lenti/BCAR-FG virus directly into each well and mix gently.
  f. On Day 3, harvest all cells into a 15-mL tube.
  g. Spin at 300 × *g* for 5 min, aspirate the supernatant fully.
  h. Resuspend in fresh C10 medium and replate in 24-well format.
  i. Again, supplement with IL-7 and IL-15 at 10 ng/mL each.
    **CRITICAL:** One day after viral transduction, replace the culture medium with fresh C10 medium to remove residual virus.
  j. On Day 7, take a small aliquot of BCAR-NKT/FG cells for FACS staining to assess transduction rate and phenotype using the following antibodies: Fixable Viability Dye eFluor506 (Affymetrix eBioscience, cat. no. 65-0866-14, 1:500 dilution); Pacific Blue-conjugated anti-CD3 (Biolegend, cat. no. 300329, 1:500 dilution); PE/Cy7-conjugated anti-CD4 (Biolegend, cat. no. 317414, 1:400 dilution); APC/Cy7-conjugated anti-CD8 (Biolegend, cat. no. 344714, 1:300 dilution); PE-conjugated anti-human invariant NK T cell (BD Biosciences, cat. no. 552825, 1:10 dilution); Goat anti-mouse IgG F(ab’)2 secondary antibody, Biotin (Thermo Fisher, cat. no. 31803, 1:50 dilution); and APC-conjugated Streptavidin (Biolegend, cat. no. 405207, 1:1000 dilution).
    ***Note:*** Additional markers may be included as needed.
5. Restimulation of BCAR-NKT/FG cells with donor-matched irradiated a-GalCer-PBMCs.
  a. On Day 7, thaw the frozen negative fraction in a 37°C water bath.
  b. Move the cells to a 50-mL conical tube with 10 mL of pre-warmed C10 medium.
  c. Centrifuge the cells at 300 × *g* for 5 min and completely remove the supernatant.
  d. Resuspend cell pellet in 5 mL C10 medium with 5 μg/mL αGC (1–2 × 10^8^ cells/5 mL medium) and incubate at 37°C for 1 h.
  e. Irradiate cells at 6000 rads.
  f. Collect all BCAR-NKT/FG cells into a 50-mL conical tube.
  g. Determine the cell numbers of the irradiated αGC-loaded PBMCs and BCAR-NKT/FG cells using a hemacytometer.
  h. Transfer all BCAR-NKT/FG cells and the same number of αGC-loaded PBMC cells (1:1 ratio) to a 50-mL conical tube.
  i. Centrifuge the cells at 300 × *g* for 5 min and completely remove the supernatant.
  j. Resuspend the pellet in C10 medium and plate the cells at 2 × 10^6^ cells in 2 mL per well in a 24-well plate.
  k. Supplement the culture with IL-7 and IL-15 to achieve a final concentration of 10 ng/m.
  l. Continue the cell culture for the next 7 days.
  m. On Day 14, collect all BCAR-NKT/FG cells.
    **CRITICAL:** As cells reach saturation, add media with IL-7 and IL-15 at 10 ng/mL and split culture.
    ***Note:*** Cells expand 5–10 fold again and reach ∼95% NKT in the second week. Troubleshooting 2.
    ***Note:*** Cell expansion can be continued for another week.
  n. Take a small aliquot of BCAR-NKT/FG cells for flow cytometry to assess transduction efficiency and phenotype. Cells were stained with the following antibodies and analyzed using the MACSQuant 10 flow cytometer: Fixable Viability Dye eFluor506 (Affymetrix eBioscience, cat. no. 65-0866-14, 1:500 dilution); Pacific Blue-conjugated anti-CD3 (Biolegend, cat. no. 300329, 1:500 dilution); PE/Cy7-conjugated anti-CD4 (Biolegend, cat. no. 317414, 1:400 dilution); APC/Cy7-conjugated anti-CD8 (Biolegend, cat. no. 344714, 1:300 dilution); PE-conjugated anti-human invariant NK T cell (BD Biosciences, cat. no. 552825, 1:10 dilution); Goat anti-mouse IgG F(ab’)2 secondary antibody, Biotin (Thermo Fisher, cat. no. 31803, 1:50 dilution); and APC-conjugated Streptavidin (Biolegend, cat. no. 405207, 1:1000 dilution).
    ***Note:*** Additional markers may be included as needed.

### *In vivo* PK/PD study of BCAR-NKT/FG cells in a human MM xenograft NSG mouse model

**Timing: 1–2 months**

This protocol outlines the *in vivo* PK/PD study of BCAR-NKT/FG in an NSG mouse model with or without human MM xenografts. The method can be adapted for other antigen-targeting CAR-NKT cells and other cell types such as CAR-T cells and CAR-engineered MAIT cells. This protocol can also be adjusted accordingly for different tumor models ranging from blood cancers, such as acute myeloid leukemia, to solid tumors, such as melanoma and lung cancer.

6. Acclimate 6-week old NSG mice (The Jackson Laboratory, strain. no. 005557) for at least 7 days prior to immunization.

***Note:*** Two groups of mice (3–5 mice each) are required for this study.

7. Revive human MM cell line MM.1S and perform daily splitting at ∼80% confluency to expand to the necessary cell number.

***Note:*** The recommended cell count for i.v. injection of MM.1S is 1 × 10^6^ cells/mouse.

***Note:*** Skip to step 5 if studying the PK/PD of BCAR-NKT/FG cells in tumor-free models.

8. Tumor cell preparation.
  a. On the day of tumor inoculation, collect all MM.1S cells into a 50-mL conical tube and count live cells using trypan blue exclusion with a hemacytometer.
  b. Move cells for injection to a 50-mL conical tube.
    ***Note:*** Continue culturing the residual cells.
    **CRITICAL:** Prepare extra cells.
  c. Centrifuge cells at 300 × *g* for 5 min and completely remove the supernatant.
  d. Resuspend cell pellet in 1× PBS at 1 × 10^6^ cells/100 μL.
  e. Move all cells into a 1.5-mL Eppendorf tube.
    **CRITICAL:** Keep cells on ice before injection to preserve viability.
9. Tumor inoculation.
  ***Note:*** This protocol outlines the intravenous (i.v.) tail vein injection of MM.1S cells. Other injection methods, including intraperitoneal (i.p.) injection and subcutaneous (s.c.) injection, can be used for different tumor models.
  a. Take prepared MM.1S cells set on ice to vivarium as quick as possible.
  b. Prior to injection, warm the mice for 5 min using an overhead heat lamp to dilate the veins.
    **CRITICAL:** Take extra care to the mice to prevent overheating them.
  c. Resuspend cells with a pipette each time before loading the cells.
  d. Load 100 μL cells into a 0.5-mL syringe with fixed 28g needle (Exel International, cat. no. 76290-402) with no air bubbles.
  e. Restrain the mouse in an appropriately sized restrainer.
  f. Clean the tail with 70% ethanol using a swab.
  g. Using the non-dominant hand, stabilize and straighten the tail.
  h. Locate the vein on one side of the tail at mid-length.
  i. Using the dominant hand, inject 100 μL of MM.1S cells into the vein towards the direction of the head, keeping the needle and syringe parallel to the tail.
    **CRITICAL:** Inject the cells slowly.
    **CRITICAL:** There should be no resistance when depressing the plunger. If resistance is felt, remove the needle and reinsert it at a new site.
  j. Remove the needle and apply gentle compression until bleeding stops.
  k. Return mice to their cage and observe to ensure that bleeding does not resume.
10. BCAR-NKT/FG cell preparation on Day 0.
  ***Note:*** Time points for effector cell injection may carry with the tumor model.
  a. On the day of effector cell injection, collect all BCAR-NKT/FG cells into a 50-mL conical tube and count live cells using trypan blue exclusion with a hemacytometer.
  b. Move the selected cell quantity into a separate 50-mL conical tube.
    ***Note:*** Residual cells can continue to be cultured.
    **CRITICAL:** Prepare extra cells.
  c. Centrifuge cells at 300 × *g* for 5 min and completely remove the supernatant.
  d. Resuspend cell pellet in 1× PBS at 10 × 10^6^ cells/100 μL 1× PBS.
  e. Move all cells into a 1.5-mL Eppendorf tube.
    **CRITICAL:** Keep cells on ice before injecting to preserve viability.
11. BCAR-NKT/FG cell injection on Day 0.
  ***Note:*** This protocol outlines the retro-orbital (r.o.) injection of BCAR-NKT/FG cells. Effector cells can also be injected using an i.v. tail vein method.
  a. Take prepared BCAR-NKT/FG cells set on ice to vivarium as quick as possible.
  b. Anesthetize the mice with isoflurane chamber.
  c. Resuspend cells with a pipette each time before loading the cells.
  d. Load 100 μL cells into a 0.5-mL syringe with fixed 28 g needle (Exel International, cat. no. 76290-402) with no air bubbles.
  e. Position the mouse on its side and restrain it with the non-dominant hand. Use the thumb to retract the skin above and below the eye.
  f. With the dominant hand, insert 1/4 to 1/3 of the needle, bevel down, at a 45° angle through the medial canthus.
  g. Slowly inject 100 μL of BCAR-NKT/FG cells into the retro-bulbar sinus.
    ***Note:*** A degree of resistance is expected as the eye moves back into the sinus.
  h. Remove the needle gently to avoid injury to the eye.
  i. Return mice to their cage and monitor them during the recovery process.
12. BLI monitoring (2–3 times per week). Troubleshooting 4 and 5.
  a. Anesthetize the mice using an isoflurane chamber with 2% isoflurane for both induction and maintenance, and an oxygen flow rate of 2 L/min.
  b. Perform i.p. injection of 3 mg D-luciferin (Caliper Life Science, cat. no. XR-1001) per mouse.
  c. Five min after injection, acquire live animal imaging using the Spectral Advanced Molecular Imaging (AMI) HTX imaging system, with a 90-s exposure time and binning set to 2 × 2.
    **CRITICAL:** A 5-min incubation is required for luciferin to circulate.
  d. Define Regions of Interest (ROIs) manually by outlining a square area from the head to the base of the tail for each mouse.
  e. Return mice to their cage and monitor them during the recovery process.
    ***Note:*** Bioluminescence was quantified using Aura Imaging Software. Quantification was based on total flux (photons/sec) using Aura Imaging Software (version 4.0.8) with default settings. Autofluorescence was not subtracted using background ROIs.

### *In vivo* tissue biodistribution of BCAR-NKT/FG cells in a human MM xenograft NSG mouse model

**Timing: 1 day**

***Note:*** Tissue biodistribution of effector cells should be assessed on the final day of the *in vivo* PK/PD study. This protocol can also be applied to assess other effector cells in different tumor models.

13. Mouse preparation.
  a. Anesthetize the mice using an isoflurane chamber with 2% isoflurane for both induction and maintenance, and an oxygen flow rate of 2 L/min.
  b. Perform i.p. injection of 10 mg D-luciferin (Caliper Life Science, cat. no. XR-1001) per mouse.
  c. Incubate for 5 min.
  d. Euthanize the mice and perform secondary killing.
14. Tissue collection and BLI assessment.
  a. Immediately dissect mice and collect all relevant tissue.
  b. Rinse the tissues with 1× PBS and dry on a paper towel.
  c. Place the tissues on a black paper.
  d. Acquire imaging using the Spectral AMI HTX imaging system, using the same exposure time, binning settings, and analysis parameters as described above.

## Expected outcomes

**Figure 1:**
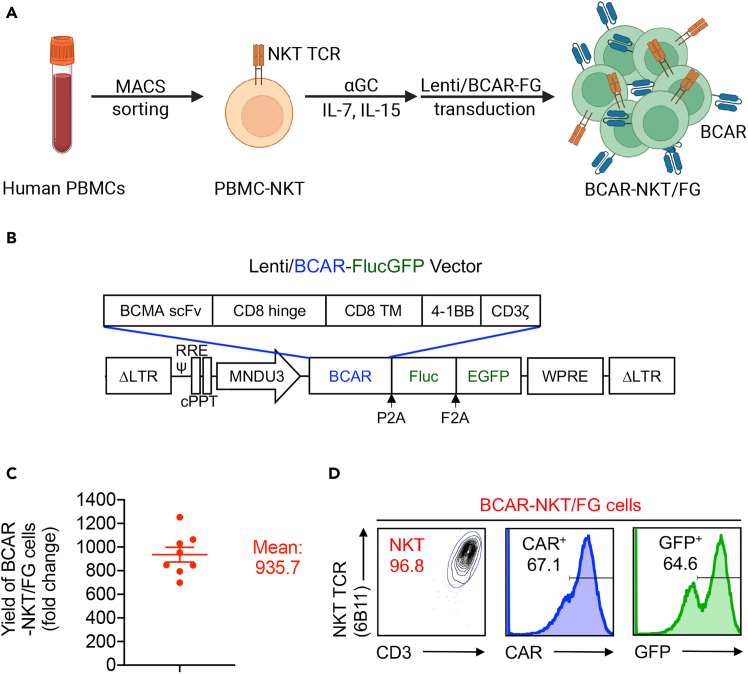
Generation and characterization of healthy donor PBMC-derived BCAR-NKT/FG cells (A) Diagram showing the generation of healthy donor PBMC-derived BCAR-NKT/FG cells. (B) Schematics showing the design of Lenti/BCAR-FlucGFP vectors. (C) Yield of BCAR-NKT/FG cells on day 14 (n = 8, n indicates different PBMC donors). Data represent mean ± SEM. (D) FACS detection of CAR and FG expressions on BCAR-NKT/FG cells. BCAR-NKT/FG cells were pre-gated on e506 viability dye-negative populations to identify live cells. CAR and GFP expression levels were then analyzed and compared to NKT cells derived from the same healthy donor PBMCs without lentivector engineering.

This protocol details the generation and assessment of PBMC-derived BCAR-NKT/FG cells (Figures 1A and 1B). Following two rounds of αGC stimulation and lentiviral transduction, NKT cells are expected to expand ∼1,000-fold (Figure 1C). Final cell products typically reach ∼95% iNKT TCR expression and display high CAR and FG transduction efficiency, as confirmed by flow cytometry (Figure 1D).

**Figure 2:**
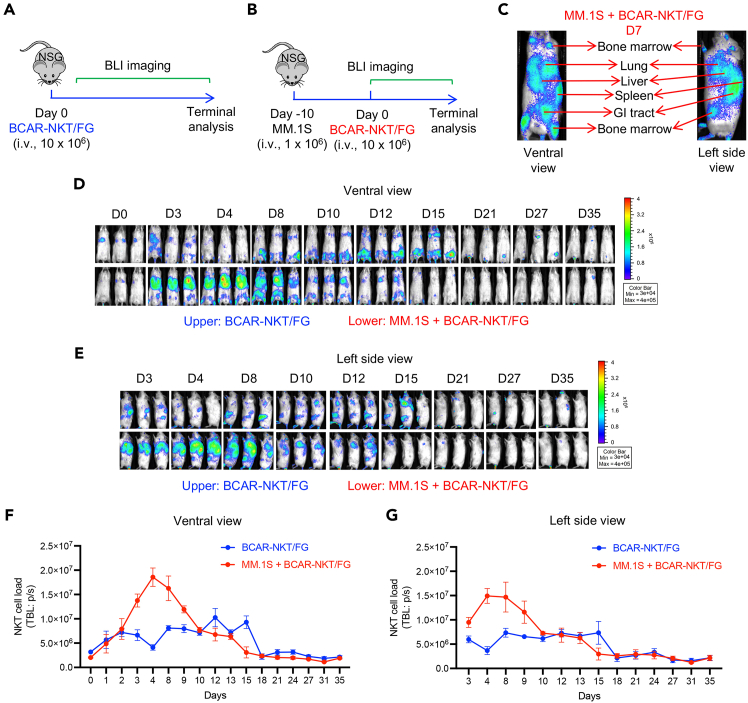
*In vivo* PK/PD study of BCAR-NKT/FG cells in tumor-free and human MM.1S-bearing NSG mice (A and B) Schematic of the BLI-based PK/PD study. BCAR-NKT/FG cells were administered intravenously in tumor-free NSG mice (A), or in human MM.1S-bearing NSG mice. The systemic distribution of BCAR-NKT/FG cells was monitored by BLI over time. (C) Representative BLI images from MM.1S-bearing mice at Day 7 post-BCAR-NKT/FG infusion, highlighting signal localization in bone marrow, lung, liver, spleen, gastrointestinal (GI) tract, and other tissues. (D and E) Serial BLI images of tumor-free (upper panels) and MM.1S-bearing (lower panels) mice from ventral (D) and left side (E) views at indicated time points. (F and G) Quantification of total body luminescence (TBL) signal over time from ventral (F) and left side (G) views. Data represent mean ± SEM (n = 3 mice/group).

This protocol also enables *in vivo* tracking of BCAR-NKT/FG cells using BLI (Figures 2A–2C). In tumor-free NSG mouse models, BLI performed at multiple time points reveals the dynamic systemic distribution and gradual clearance of BCAR-NKT/FG cells (Figures 2A and 2D–2G). The cells are expected to home to various tissues including the lungs, spleen, liver, bone marrow, and gastrointestinal (GI) tract, reflecting their innate tissue-trafficking profile in the absence of tumor cues (Figures 2D–2G). In human MM xenograft NSG mouse models, BCAR-NKT/FG cells exhibit robust expansion within tumor-associated tissues, particularly the lungs, liver, and bone marrow, indicating antigen-driven stimulation and proliferation at disease sites (Figures 2B and 2D–2G).

**Figure 3:**
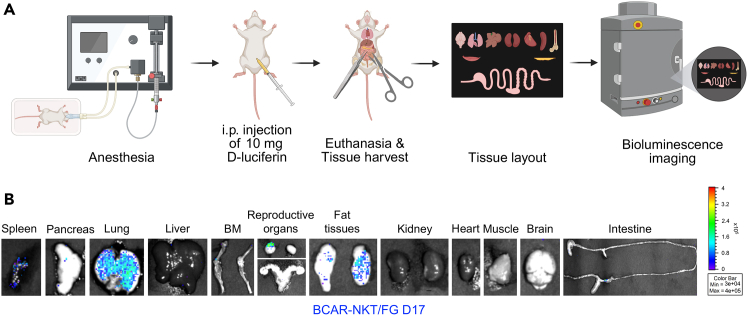
*In vivo* tissue biodistribution of BCAR-NKT/FG cells in tumor-free and human MM.1S-bearing mice (A) Schematic workflow of terminal tissue-level biodistribution analysis. (B) Representative BLI images of individual tissues from a tumor-free NSG mouse on Day 17 following BCAR-NKT/FG cell infusion.

In addition, terminal tissue-level BLI confirms the biodistribution and regional accumulation of BCAR-NKT/FG cells at the study endpoint (Figure 3A). In tumor-free mice analyzed on Day 17, signal is detected in the lungs, fat tissues, reproductive organs, bone marrow, pancreas, and spleen (Figure 3B), consistent with the broad but selective tissue tropism of NKT cells.

Altogether, this protocol allows for in-depth characterization of PBMC-derived BCAR-NKT cell kinetics and tissue targeting in both physiological and pathological contexts. It serves as a robust tool for evaluating CAR-NKT cell trafficking, persistence, and tumor engagement, and can be further adapted for solid tumor models and other engineered immune effector cell types.

## Quantification and statistical analysis

All statistical analyses were conducted using GraphPad Prism 8. For comparisons between two groups, a two-tailed Student’s t-test was applied. When comparing more than two groups, ordinary one-way ANOVA followed by Tukey’s post hoc test was used. Unless noted otherwise, data are displayed as mean ± SEM. In figures and legends, “n” indicates the number of independent biological replicates. Statistical significance was defined as p < 0.05. ns, not significant; ∗p < 0.05; ∗∗p < 0.01; ∗∗∗p < 0.001; ∗∗∗∗p < 0.0001.

## Limitations

While BLI offers non-invasive tracking, its resolution is limited for deep tissues or small cell populations. Signal intensity may not linearly correlate with absolute cell numbers, especially in dense or highly vascularized tissues like the liver and lungs.

The NSG mouse model lacks a fully functional immune system, which facilitates human cell engraftment but does not fully recapitulate the complexity of an intact immune environment. Interactions between CAR-NKT cells and endogenous immune compartments including regulatory T cells or myeloid-derived suppressor cells cannot be evaluated in this model.

This protocol is designed to evaluate CAR-NKT cell pharmacokinetics and biodistribution rather than therapeutic efficacy; therefore, tumor burden tracking methods are not included and should be incorporated separately when conducting efficacy studies.

## Troubleshooting

### Problem 1

Low NKT cell yield after initial MACS enrichment. Related to “generation of human PBMC-derived BCAR-NKT/FG cells” step (2).

### Potential solution

Low yield may result from poor PBMC quality or a low baseline iNKT frequency. To improve recovery, consider performing a second MACS round, using freshly isolated PBMCs when possible, or increasing the starting PBMC input.

### Problem 2

Poor expansion during αGC stimulation. Related to “generation of human PBMC-derived BCAR-NKT/FG cells” step (3 and 5).

### Potential solution

Suboptimal expansion can result from insufficient stimulation by irradiated PBMCs or cytokine concentration. Ensure complete αGC loading and that PBMCs are freshly irradiated. Verify the activity and concentration of IL-7 and IL-15 used in the culture.

### Problem 3

Low CAR transduction efficiency. Related to “generation of human PBMC-derived BCAR-NKT/FG cells” step (4).

### Potential solution

Low transduction rates may stem from poor lentiviral quality or suboptimal cell conditions. Use high-titer lentivirus and optimize the timing and conditions of transduction, including fresh media and healthy, actively dividing cells. Spinfection may also be considered to enhance transduction rates.

### Problem 4

Weak or diffuse BLI signal *in vivo*. Related to “in vivo PK/PD study of BCAR-NKT/FG cells in a human MM xenograft NSG mouse model” step (12).

### Potential solution

This issue may be caused by inconsistent D-luciferin administration or suboptimal imaging timing. Confirm proper intraperitoneal injection of luciferin and allow 5–10 min for substrate distribution before acquiring BLI images.

### Problem 5

Loss of signal or rapid clearance of cells *in vivo*. Related to “in vivo PK/PD study of BCAR-NKT/FG cells in a human MM xenograft NSG mouse model” step (12).

### Potential solution

This may occur due to poor cell viability at the time of injection. Ensure that infused cells have >90% viability, keep them on ice prior to injection, and minimize handling time to preserve cell function and persistence.

## Resource availability

### Lead contact

For additional details or requests for materials, please contact the lead contact, Lili Yang (liliyang@ucla.edu), who will provide the necessary resources.

### Technical contact

For direct technical inquiries regarding this protocol, please contact the technical contact, Yan-Ruide Li (charlie.li@ucla.edu), who will answer all the questions.

### Materials availability

Human tumor cell lines generated in this study will be made available upon reasonable request.

### Data and code availability

This study did not generate new datasets or code.

## Acknowledgments

We thank the University of California, Los Angeles (UCLA) CFAR Virology Core for providing human cells. This work was supported by a Partnering Opportunity for Discovery Stage Award from the 10.13039/100000900California Institute for Regenerative Medicine (DISC2-13505 to L.Y.), a 10.13039/100005595UCLA BSCRC Stem Cell Research Innovation Award (to L.Y.), and an Ablon Scholars Award (to L.Y.). L.Y. is an investigator of the Parker Institute for Cancer Immunotherapy (PICI) at UCLA. Y.-R.L. is a postdoctoral fellow supported by a 10.13039/100005595UCLA MIMG M. John Pickett Post-Doctoral Fellow Award, a CIRM-BSCRC Postdoctoral Fellowship, a 10.13039/100005595UCLA Sydney Finegold Postdoctoral Award, and a UCLA Chancellor’s Award for Postdoctoral Research. Graphical abstract and some figures were created using Biorender.com.

## Author contributions

Z.L., Y.-R.L., and L.Y. designed the experiments, analyzed the data, and wrote the manuscript. Y.-R.L. and L.Y. conceived and oversaw the study. Z.L. performed the experiments and wrote the manuscript.

## Declaration of interests

Y.-R.L. and L.Y. are inventors on patents relating to this manuscript. L.Y. is a scientific advisor to AlzChem and Amberstone Biosciences and a co-founder, stockholder, and advisory board member of Appia Bio. None of the declared companies contributed to or directed any of the writing of this manuscript.
